# A novel *MYOC* heterzygous mutation identified in a Chinese Uygur pedigree with primary open-angle glaucoma

**Published:** 2012-07-18

**Authors:** Su-ping Cai, Paerheti Muhemaiti, Yan Yin, Hongbo Cheng, A. Di Ya, Maliyamu Keyimu, Xu Cao, Ning Fan, Liqiong Jiang, Naihong Yan, Xiaomin Zhou, Yun Wang, Xuyang Liu

**Affiliations:** 1 Shenzhen Eye Hospital, Jinan University, Shenzhen, P.R.China; 2 A Di Ya Eye Hospital, Wulumuqi, Xinjiang, P.R.China; 3 Ophthalmic Laboratories, Translational Neuroscience Center, West China Hospital, Sichuan University, P.R.China

## Abstract

**Purpose:**

To characterize the clinical features of a Chinese Uygur pedigree with primary open-angle glaucoma (POAG) and to identify mutations in two candidate genes, trabecular meshwork inducible glucocorticoid response (*MYOC*/*TIGR*) and human dioxin-inducible cytochrome P450 (*CYP1B1*).

**Methods:**

Twenty one members from a Chinese Uygur family of four generations were included in the study. All participants underwent complete ophthalmologic examinations. Five were diagnosed as POAG, four as glaucoma suspects, and the rest were asymptomatic. Molecular genetic analysis was performed on all subjects included in the study. All exons of *CYP1B1* and *MYOC* were amplified by polymerase chain reaction (PCR), sequenced and compared with a reference database. The variations detected were evaluated in available family members as well as 102 normal controls. Possible changes in structure and function of the protein induced by amino acid variance were predicted by bioinformatics analysis.

**Results:**

Elevated intraocular pressure and late-stage glaucomatous cupping of the optic disc were found in five patients of this family. A novel heterozygous missense mutation c.1151 A>G in exon 3 of *MYOC* was found in all five patients diagnosed as POAG and four glaucoma suspects, but not in the rest of the family members and 102 normal controls. This mutation caused an amino acid substitution of aspartic acid to glycine at position 384 (p. D384G) of the MYOC protein. This substitution may cause structural and functional changes of the protein based on bioinformatics analysis. No mutations were found in *CYP1B1*.

**Conclusions:**

Our study suggests that the novel mutation D384G of *MYOC* is likely responsible for the pathogenesis of POAG in this pedigree.

## Introduction

Glaucoma is one of the leading causes of irreversible blindness in the world and is a neurodegenerative disorder characterized by progressive loss of retinal ganglion cells that results in the excavation of the optic disc and gradual constriction of visual field [1]. It is usually associated with elevation of intraocular pressure (IOP) [2]. The most prevalent form of glaucoma is primary open-angle glaucoma (POAG; OMIM 137760) [3]. POAG has two forms: juvenile and adult onset, with the latter most commonly seen. Juvenile open-angle glaucoma (JOAG) may manifest clinically between the ages of 3 and 30 [4,5], while adult POAG usually occurs after the age of 40 [6,7]. Although the exact mechanisms of POAG are not fully understood, it is generally accepted that genetic factors play an important role in its pathogenesis. About 30%–56% of patients with glaucoma or ocular hypertension (OHT) have a positive family history; first-degree relatives of POAG patients are seven to ten times more likely to have POAG [8,9]. Four genes, including trabecular meshwork inducible glucocorticoid response (*MYOC*), human dioxin-inducible cytochrome P450 (*CYP1B1*), optineurin (*OPTN*), and *WD* repeat domain 36 (*WDR36*), have been identified as glaucoma-associated genes [10]. Up to date, more than 70 mutations have been detected in *MYOC*, the first identified POAG causing gene, worldwide [11,12]. About 90% of the mutations were located in exon 3 of *MYOC* where the olfactomedin-like domain is located [13]. Of particular interest, the *MYOC* gene has been reported to interact with *CYP1B1* through a digenic mechanism, leading to JOAG [14-16]. Both *MYOC* and *CYP1B1* consist of three exons, but in *CYP1B1*, only exon 2 and 3 encode the protein. In this study, both *MYOC* (three exons) and *CYP1B1* (exon 2 and 3) genes were analyzed.

## Methods

### Family recruitment and clinical examination

**Figure 1:**
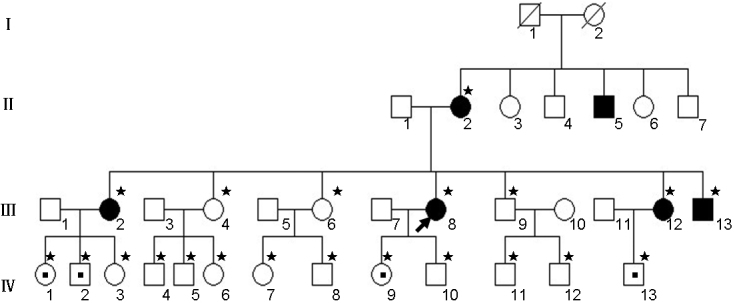
Pedigrees of the Chinese Uygur family with primary open-angle glaucoma. Filled squares and circles are affected males and females, respectively. Small filled square in the middle of the circle or square indicates the carrier. Arrowhead indicates the proband. The asterisks indicate the individuals who had undergone clinical examination. Molecular analyses were done in all individuals marked with asterisks.

A four-generation pedigree with POAG (Figure 1) was recruited from A Di Ya Eye Hospital (Wulumuqi, Xinjiang, P. R. China). No consanguineous marriage was noticed in the family. Twenty one members of the family underwent complete ophthalmologic examinations including slit-lamp biomicroscopy, gonioscopy, IOP measurement (Canon TX-F Non-contact tonometer; Canon Inc., Tokyo, Japan), fundus examination and visual field test. All individuals in the control group were healthy and with no history of other familial inherited diseases. The study was approved by the Medical Ethics Committee of the Shenzhen Eye Hospital of Jinan University in Guangdong Province, Shenzhen. Informed consent was obtained from all participants according to the principles of Declaration of Helsinki. All subjects were clinically evaluated by glaucoma specialists.

### Mutation screening and sequence analysis

Genomic DNA was extracted from 200 μl venous blood using a Qiamp Blood Kit (Qiagen, Hilden, Germany). All the procedures were performed according to the manufacturer’s instructions. DNA integrity was detected by 1% agarose gel electrophoresis. Intronic primers flanking the exons were designed (Table 1) based on gene sequences of *MYOC* (GenBank AF001620) and *CYP1B1* (GenBank U56438), and synthesized by Invitrogen (Carlsbad, CA). Exons of *MYOC* and *CYP1B1* were amplified by PCR using the designed forward and reverse primers. PCR amplification was conducted in a MyCycler thermocycler (Bio-Rad, Hercules, CA). The 30 μl PCR reaction mixtures included 30–40 ng genomic DNA, 1.0 μM of each of the forward and reverse primers, and 15 μl of 2× Taq Master Mix (inculding1× PCR buffer, 2.5 mM MgCl**_2_**, 0.3 mM of each of dNTPs, 1.5 U Pfu DNA polymerase). All reagents used in this procedure were purchased from SinoBio Biltech Co. Ltd, Shanghai, China. The cycling conditions included an initial denaturation at 95 °C for 5 min, followed by 35 cycles of denaturation at 95 °C for 30 s, annealing at 58.4 °C for 30 s (the second exon of *CYP1B1* at 55 °C*)*, extension at 72 °C for 60 s (the second exon of *MYOC* for 30 s and the rest for 90 s), and then a final extension at 72 °C for 5 min. The amplified products were purified with a cycle-pure kit (OMEGA; Bio-Tek, Doraville, GA) and sequenced on the ABI 3730XLautomated DNA sequencer (Applied Biosystems, Foster City, CA). Sequence data were compared pair-wise with the published *MYOC* and *CYP1B1* sequences. Mutation was named according to the nomenclature recommended by the Human Genomic Variation Society (HGVS).

**Table 1:** Primers used in PCR for amplification of *MYOC* and *CYP1B1*.

**Exons**	**Primer sequence (forward/reverse)**	**Product size (bp)**
*MYOC* 1	PF 5′-CCAAACAGACTTCTGGAAGG-3′	904
	PR 5′-TAGCAGGTCACTACGAGCC-3′	
*MYOC* 2	PF 5′-TGTCATCCTCAACATAGTCA-3′	351
	PR 5′-TTCTGTTCCTCTTCTCCTC-3′	
*MYOC* 3	PF 5′-CCAGGGCTGTCACATCTACT-3′	933
	PR 5′-CATCTCCTTCTGCCATTGC-3′	
*CYP1B1* 2	PF 5′-CATTTCTCCAGAGAGTCAGC-3′	1260
	PR 5′-GCTTGCAAACTCAGCATATTC-3′	
*CYP1B1* 3	PF 5′-ACCCAATGGAAAAGTCAGCC-3′	927
	PR 5′-GCTTGCCTCTTGCTTCTTATT-3′	

### Bioinformatics analysis

The Clustalw tool was used to align the protein sequences among eight different species. The possible functional impact of an amino acid change was predicted by Polyphen and Sorting Intolerant From Tolerant (SIFT). Garnier-Osguthorpe-Robson (GOR IV) software was used to predict the effect of the mutation on the secondary structure of MYOC [17]. This method infers the secondary structure of a sequence by calculating the probability for each of the four structure classes (helix, sheet, turn, and loop) based on the central residue and its neighbors from the calculated matrices.

## Results

### Clinical findings of the pedigree

**Figure 2:**
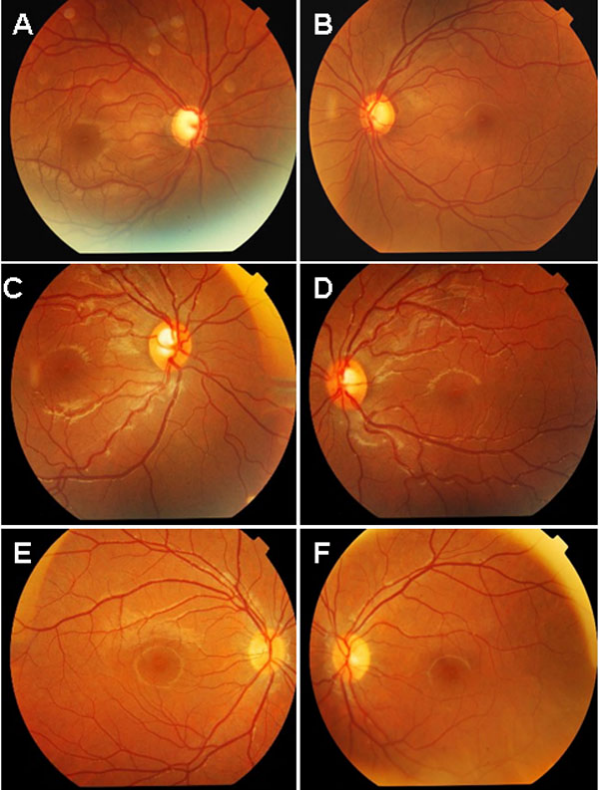
Fundography of glaucoma patients and suspects. Fundography of a glaucoma patient III-12 (**A**, **B**), two suspects: IV:9, a 9-year-old (**C**, **D**), and IV:13, a 4-year-old (**E**, **F**).

Five out of twenty one members examined in this four-generation family were found to have elevated intraocular pressure and late-stage glaucomatous cupping of the optic disc. Four suspects were found to have elevated intraocular pressure without characteristic glaucomatous optic disc changes (Table 2, Figure 2). The proband (III-8, a thirty-three-year-old female) was diagnosed as JOAG in both eyes at the age of 28, with elevated IOPs (42 mmHg OU), open anterior chamber angle, enlarged cup disc ratio of 0.95/0.8 (OD/OS). No systemic disorders were found. Trabeculectomy was performed for both eyes at the age of 30. Three years later, IOP measured 15~18 mmHg OS. The visual acuity in the right eye worsened to no light perception. The proband’s mother (II-2) was diagnosed as POAG at the age of 55. She recalled that she had reduced vision since her early thirties and became totally blind at her early forties. Her IOPs measured 48~50 mmHg OU with the cup/disc ratio of 1.0 OU. Both eyes of the proband’s uncle (II-5) became blind when he was in his early 40s. Since his blood sample was not available, this patient was not included in the study. The proband’s older sister (III-2) was diagnosed as JOAG at the age of 40 with IOPs of 42/50 mmHg (OD/OS). Funduscopy revealed a glaucomatous excavation and atrophy of the optic disc in both eyes with a cup-disc ratio of 0.95/1.0 (OD/OS). She recalled that she had reduced vision in her early thirties. Patient III-12 was diagnosed as JOAG in both eyes at the age of 27. Her IOPs was 35 mmHg OS and 32 mmHg OD. A cup/disc ratio of 0.8/0.8 OU was noticed in the patient (Figure 2). Patient III-13 was diagnosed as JOAG at the age of 25 with the visual acuity of light perception OD, and hand motions OS. His maximal IOPs measured 52 mmHg OU. Funduscopy revealed optic nerve atrophy in both eyes with a cup-disc ratio of 1.0. Patient IV:1, a 22-year-old female, was a glaucoma suspect. Her IOP measured 21/24mmHg (OD/OS) and no defect in optic disc and visual field was noticed. Her 20-year-old younger brother (IV:2) had elevated IOP of 24 mmHg OD and 26 mmHg OS with normal optic disc and visual field. Two young family members, one was a 9-year-old girl (IV:9), the other was a 4-year-old boy (IV:13), were found to have elevated IOP. Possible optic disc damage was found in the 9-year-old girl but was not found in the 4-year-old boy. The visual field results of both of them were unreliable. No ocular abnormalities were found in the rest of the family numbers.

**Table 2:** Patient data from this POAG pedigree.

**Patient**	**Sex**	**Age at study (years)**	**Diagnosis age (years)**	**Maximal IOP (mmHg)**	**C/D ratio**	**Operation age (years)**
II-2	female	60	55	48~50 (ou)	1.0/1.0	—
III-2	female	40	40	42 (od), 50 (os)	0.95/1.0	—
III-8	female	33	28	42 (ou)	0.95/0.8	30
III-12	female	29	27	32 (od), 35 (os)	0.8/0.8	—
III-13	male	25	25	52 (ou)	1.0/1.0	—
IV:1	female	22	22	21 (od), 24 (os)	0.4/0.4	—
IV:2	male	20	20	24 (od), 26 (os)	0.4/0.4	—
IV:9,	female	9	9	25 (od), 28 (os)	0.4/0.4	—
IV:13	male	4	4	25 (od), 25 (os)	—*	—

* indicates that C/D ratio was not able to be obtained since the child was not cooperative with exam.

### *MYOC* and *CYP1B1* mutation identification and analysis

**Figure 3:**
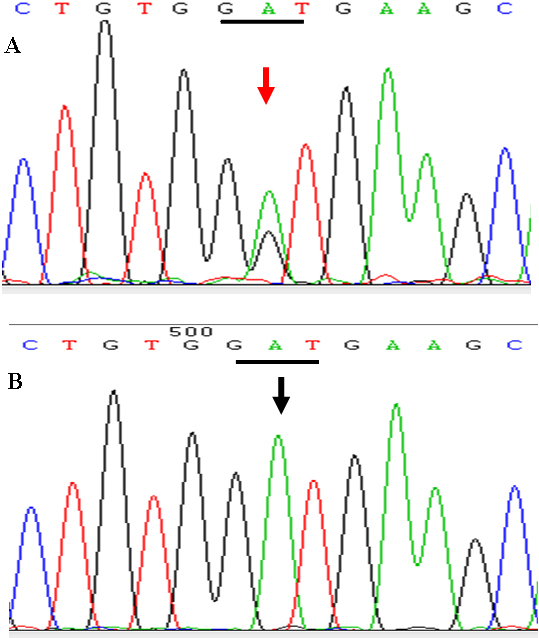
Sequencing results of the *MYOC* gene. **A**: A heterozygous A to G transversion at codon 384 in one patient from the family (red arrow). **B**: Wild type sequence from an unaffected member.

Sequence analysis of *MYOC* revealed a heterozygous missense mutation, c.1151 A>G (p. D384G), in exon 3 of all five patients, four glaucoma suspects. No such mutation was detected in other asymptomatic members of the family and 102 normal control subjects included in this study (Figure 3). No other mutations were identified in this gene and in *CYP1B1*.

### Bioinformatic analysis

**Figure 4:**
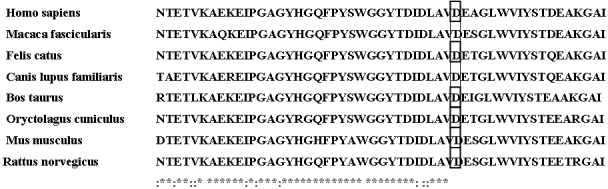
The mutation involved a highly conserved residue. The aspartic acid at position 384 are highly conserved for MYOC, which was demonstrated by analysis of orthologs from eight different species.

**Figure 5:**
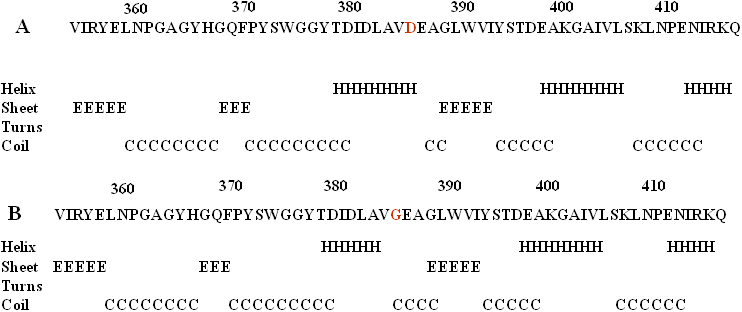
The effect of p. D384G on the secondary structure of MYOC using the GORIV method. **A**: The secondary structure of wild type MYOC around the site D384. **B**: The secondary structure of mutant G384 of MYOC of the corresponding region.

The mutation c.1151 A>G in exon 3 of *MYOC* would result in replacement of aspartic acid by glycine. Aspartic acid in 384 was highly conserved for MYOC based on analysis of orthologs from eight different species using the online Clustalw tool (Figure 4). The pD384G mutation of MYOC was predicted to be “probably damaging” by Polyphen with a score of 2.189 and “affect protein function” by SIFT with a score of 0.00, which indicated that protein function was affected by amino acid changes (score <0.05 indicating that the amino-acid change may affect the protein function).The results for secondary structure prediction by the GORIV method suggested that the mutant MYOC 384G replace two β sheet “E”s with two coil “C”s at amino acid 384 and 385 (Figure 5).

## Discussion

*MYOC* was the first identified gene associated with pathogenesis of POAG [11]. Previous studies showed that *MYOC* mutations exist in a greater proportion of JOAG and nearly 3% of adult onset POAG patients [18,19]. *MYOC* mutations have been reported in POAG pedigrees from many different ethnic groups [18-36]. In this study, the *MYOC* gene was screened for mutations in a four-generation Uygur, an ethnic minority in China, family.

The identified *MYOC* mutations involved in POAG in different ethnic populations include Pro370Leu, Gly367Arg, Arg158Gln, Gly252Arg, Arg272Gly, Asn 450 Tyr, Gln48His and Asp 384 Asn [11,20-26,35-37]. Most *MYOC* mutations were located in exon 3, while some in exon 1 [38]. In this study, an A to G transversion at the second base of codon 384 (in exon 3 of *MYOC)*, which resulted in aspartic acid to glycine amino acid substitution, was identified in all affected glaucoma patients (II:2, III:2, III:8, III:12, III:13), and four glaucoma suspects (IV:1, IV:2, IV:9, IV:13), but not in other unaffected members of the family and 102 normal control subjects. The results of GORIV suggested that p.D384G lead to a secondary structure change by replacing two β sheet “E”s with two coil “C”s around the aspartic acid residue 384, which might interfere with the correct folding of the protein. The *MYOC* gene mutation exhibited as heterozygous. To date, to the best of our knowledge, this mutation has not been reported in any other ethnic groups. The majority of POAG were juvenile onset in this pedigree since the onset of symptoms occurs before the age of 30 in most patients. Some patients became blind before they came to see glaucoma specialist. This is consistent with previous finding that *MYOC* mutations exist in a greater proportion of JOAG patients. The D384G mutation of MYOC in this family appears to be the cause of the disease in this family. In this pedigree, all the glaucoma suspects including a 22-year-old female, a 20-year-old male, a 9-year-old girl and 4-year-old boy in the fourth generation were found to harbor this mutation. They were found to have normal optic disc appearance except the 9-year-old girl who may have early stage glaucomatous optic disc. Careful follow up of these individuals will make it possible to identify new patients at the early stage. Early and proper control of their IOP should help to prevent them from having blindness at young age like the JOAG patients with the same mutation in their family. It should be noted that the Xingjiang (Uygur) are very sparsely populated. The inhabitants there usually do not come to see the doctors unless they have to or are required to do so since transportation is very inconvenient.

Recently, another gene, *CYP1B1*, has been suggested to modify the glaucoma phenotype [10]. It may act as a modifier of *MYOC* expression or the two genes may interact via a common pathway [39,40]. We also screened for *CYP1B1* gene, but no mutation was detected.

In summary, this is the first time that D384G mutation was identified in glaucoma patients. This study added a novel mutation to the existing spectrum of *MYOC* mutations, suggesting that a mutation in *MYOC* correlated with glaucoma as observed in this family. These results provide pre-symptomatic molecular diagnosis for the members of the pedigree and are useful for follow-up of the family. All carriers of the family should undergo ophthalmologic surveillance at regular intervals for the rest of their lives.
